# Doctors Described by Nurses and Patients

**Published:** 1887-03-05

**Authors:** 


### Doctors described by Nurses and Patients.

From quite a bundle of letters, we have selected the following amusing, satirical, good, bad, and indifferent definitions of a doctor, by the patients who use and the nurses who aid him.

### DREADFUL DOCTOR DRUGS.

A man of pills, of draughts, and lotions,
Concoctor of most nauseous potions.—PADDY.

### SOMETHING LIKE A DOCTOR.

A nurse describes the ideal physician thus:—A concrete embodiment of Christian charity—serving as a haven of refuge and help for suffering humanity.—TABS.

### POOR DEAR MAN.

A lady correspondent thus describes her medical attendant: Active, benevolent, comforting, decided, earnest, far-seeing, genuine, honest, intellectual, just, kind, liberal, merciful, neat, observant, prudent, quiet, refined, talented, scientific, useful, virtuous, wise, Christian, blessed, zealous, and much more is my doctor.—CONDORCET.

### FACING DEATH EVERY DAY.

To define "Doctor," droll, severe, or humorous,
Declining all, shall much prefer the serious:
Neglecting self, where contagion's danger lurks,
In hospital or home, his duty never shirks.
Doctor is he who, midst vile and squalid slums,
Visits those dens a so-called Christian shuns;
With pay but small, some gratitude, more curses,
"Thy work well done," his conscience reimburses.
PATIENT.

### OUR PROVIDENCE ON EARTH.

The doctor is the harbinger of fate,
Cometh he early or cometh he late;
He is the messenger of joy or woe,
Into whatever household he doth go.
His words, his looks, his very accents tell,
The anxious mother's heart if all be well.
The doctor is our providence on earth;
He helps to fan the spark of life at birth
He stands beside us when awaiting death,
And (as it were) delivers up our final breath.
All honour, then, to him, our trusty friend,
Our help from life's beginning to life's end.—K. M.

### DOCTOR BUSTLE.

Dr. Bustle alights, and is knocking at the door before his coachman has brought the horses to a standstill. He is impatient at the delay necessarily caused by having to wait for the door to be opened. He speeds up the stairs, not waiting for invitation, invariably makes for the blind, which he pulls up with a clatter, and then rushes at his patient, who by this time is flushed, and palpitating from his irritating presence. He asks questions in an abrupt manner (questions *the patient* wanted to ask are frightened away). Poor patient! medicine alone must cure, not Doctor Bustle's gentle, sympathising visit.—GRETTA.

### AD MEDICUM.

A craft is yours beyond the rest abstruse,
A title of most careless popular use,
That stamps you teacher. Yet how
Small the part who teach, as well
As wield the godlike art.
Cruel you must be to be kind, and break
Too oft your own good rules for others' sake.
Yet is your practice and profession one,
This without that were utterly undone.
You oftener than the priesthood hear confessions
In this our day, of follies and transgressions;
Lax-lived or strict, yet fame agrees in giving
Your craft repute as lovers of *good living*.—OSTRICH.

### MATER ON HER PHYSICIAN.

He must have taken the Degree
Which makes of him a real M.D.,
So henceforth he diagnoses,
And gives sick folks horrid doses.
Whether he's old, whether he's young,
Reticent, or with a glib tongue;
Whether dark or fair, slim or stout,
He's the man we can't do without.
He helps us first into this life,
With all its harry, pain, and strife;
Physics us in every stage,
In childhood, middle, and old age.
He lays down the law, airs his views,
Allows of this, and that tabooes,
And then, as the best doctor will,
He sends us in an awful bill.—MATER.

### WHO IS HE?

Healer and teacher both, I cannot plan
A higher ideal than this living man.
His brief, apt queries, kind but searching eye,
Take measure of the subject thoroughly.
He sees when latent valour to excite,
And steels weak Nature for the coming fight.
Synthetic, prompt to act, and swift to heal,
A grateful patient must such influence feel.
Leaving old lines when he can see the new,
Careless what cautious brethren say or do,
If any other friend these lines should scan,
I think they may divine my "living man."—A. M. E.

### A JUST TRIBUTE.

A Doctor is a gentleman of great intellect, mind, and courage, who little deserves the amount of abuse and reproach which is too frequently showered upon him by those who (to put it mildly) do not care to acknowledge his great ability and intellectual powers. It is true that he is often of a somewhat stern temperament, and sometimes appears rough and inattentive, but what we should do without him I dare not surmise. In my opinion he richly deserves a far greater amount of credit than he generally succeeds in obtaining from his not too grateful patients.—PERSEVERANCE.

### THE MOTHER'S HOPE.

Oft in the darkness of the night,
Longing for morning's friendly light,
A mother watcheth silently
Beside the restless couch of pain,
Whereon her darling lies, in vain
Trying to sleep, impatiently.
She hears a knock!—"The Doctor's here!"
At once her sorrow seems to clear,
As at the deepest dark comes dawn;
Hope in her heart soon kindles joy,
"Oh! Doctor, *you* can save my boy,
Save *me* from anguish so forlorn."—K. M.

